# Comparative Safety and Efficacy of Eight Antithrombotic Regimens for Patients With Atrial Fibrillation Undergoing Percutaneous Coronary Intervention

**DOI:** 10.3389/fcvm.2022.832164

**Published:** 2022-03-21

**Authors:** Bo Liang, Yong-Chun Zhu, Ning Gu

**Affiliations:** ^1^Nanjing University of Chinese Medicine, Nanjing, China; ^2^Nanjing Hospital of Chinese Medicine Affiliated to Nanjing University of Chinese Medicine, Nanjing, China

**Keywords:** antithrombotic regimens, atrial fibrillation, percutaneous coronary intervention, VKA, NOAC, safety and efficacy

## Abstract

**Background:**

Antithrombotic therapy for patients with atrial fibrillation undergoing percutaneous coronary intervention is facing major treatment problems in clinical practice.

**Methods:**

We firstly conducted a Bayesian network meta-analysis to study the safety and efficacy of different antithrombotic regimens. Only randomized controlled trials from PubMed, Web of Science, Cochrane Central Register of Controlled Trials, Embase, and China National Knowledge Infrastructure were included in our study. The Bayesian random-effects model was used in this study. The primary safety and efficacy outcomes were major bleeding according to the criteria of Thrombolysis In Myocardial Infarction (TIMI) and trial-defined major adverse cardiovascular events, respectively. The secondary safety outcomes were combined TIMI major and minor bleeding, trial-defined primary bleeding events, and intracranial hemorrhage. The secondary efficacy outcomes were all-cause or cardiovascular mortality, myocardial infarction, stroke, stent thrombosis, and hospitalization.

**Results:**

Total of 11,532 patients from the five randomized controlled trials were analyzed, of whom 8,426 were male. Compared with vitamin K antagonist (VKA) plus P2Y12 inhibitor, the odds ratios (95% credible intervals) for TIMI major bleeding were 1.70 (0.77–3.80) for VKA plus dual antiplatelet therapy (DAPT), 1.20 (0.30–4.60) for rivaroxaban plus P2Y12 inhibitor, 1.00 (0.25–3.90) for rivaroxaban plus DAPT, 0.76 (0.21–2.80) for dabigatran plus P2Y12 inhibitor, 0.71 (0.25–2.10) for apixaban plus P2Y12 inhibitor, 1.40 (0.52–3.80) for apixaban plus DAPT, and 1.00 (0.27–4.00) for edoxaban plus P2Y12 inhibitor. For trial-defined major adverse cardiovascular events, compared with VKA plus P2Y12 inhibitor, the odds ratios (95% credible intervals) were 1.10 (0.61–2.00) for VKA plus DAPT, 1.20 (0.45–3.70) for rivaroxaban plus P2Y12 inhibitor, 1.10 (0.38–3.20) for rivaroxaban plus DAPT, 1.10 (0.43–3.10) for dabigatran plus P2Y12 inhibitor, 1.00 (0.47–2.20) for apixaban plus P2Y12 inhibitor, 0.99 (0.46–2.20) for apixaban plus DAPT, and 1.20 (0.43–3.40) for edoxaban plus P2Y12 inhibitor. Apixaban plus P2Y12 inhibitor was the highest-ranking of safety outcomes and VKA plus P2Y12 inhibitor was the highest-ranking of efficacy outcomes other than trial-defined major adverse cardiovascular events.

**Conclusion:**

Apixaban plus P2Y12 inhibitor seems to be linked with fewer bleeding complications while retaining antithrombotic efficacy. Moreover, for most efficacy indicators, the ranking of VKA plus P2Y12 inhibitor is still very high.

**Systematic Review Registration:**

[www.crd.york.ac.uk/prospero/], identifier [CRD42020149894].

## Introduction

In the past 50 years, cardiovascular disease brings a huge burden to countries all over the world, not only economically, although great progress has been made in the prevention and management (1). The vigorous development of coronary intervention technology has reduced the mortality of ischemic heart disease to a very low level, but antithrombotic drugs, usually aspirin and a P2Y12 inhibitor dual antiplatelet therapy (DAPT), are needed after operation to reduce the ischemic events (2). We all know that patients with atrial fibrillation (AF) also need antithrombotic therapy, whether vitamin K antagonists (VKA) or non-vitamin K antagonist oral anticoagulants (NOAC), to prevent stroke (3). Such antithrombotic strategies for AF patients undergoing percutaneous coronary intervention (PCI) require the constant balance of the risks of bleeding and ischemia (4), creating difficulties for clinicians. Theoretically, in this special population, the use of triple therapy (DAPT and VKA/NOAC) may provide better cardiovascular benefit, but this limited benefit may be offset by higher bleeding risk, and discontinuation of aspirin may lead to higher rates of stent thrombosis and ischemic events (5, 6).

Many high-quality randomized controlled trials (RCTs) have compared the safety and efficacy of different antithrombotic regimens in patients with AF undergoing PCI (7–11). Lopes et al. summarized these high-quality RCTs and found that an NOAC plus a P2Y12 inhibitor without aspirin may be the most preferred antithrombotic regimen option and the favorable treatment option for most patients with AF undergoing PCI (12). Although these findings give us great enlightenment, there is still no best answer to which NOAC is the best and most dominant. Here, we conducted a Bayesian network meta-analysis which allowed simultaneous comparisons of multiple antithrombotic strategies to finally provide the latest and comprehensive evidence of antithrombotic therapy for this special population.

## Methods

We followed the Preferred Reporting Items for Systematic Reviews and Meta-Analyses (PRISMA) guidelines (13). We developed a protocol and registered on PROSPERO (CRD42020149894).

### Search Strategy and Selection Criteria

Two reviewers searched PubMed, Web of Science, Cochrane Central Register of Controlled Trials, Embase, and China National Knowledge Infrastructure for relevant published studies before 30 September 2021, without any language restrictions. We used keywords related to atrial fibrillation, AF, percutaneous coronary intervention, PCI, acute coronary syndrome, ACS, NOAC, VKA, antithrombosis, and antiplatelets. We only included RCTs that compared different antithrombotic strategies in patients with AF undergoing PCI.

The inclusion criteria of our study included (1) RCTs with at least two comparators; (2) study patients were AF, including previous, persistent, permanent, or paroxysmal AF, and had undergone PCI; (3) patients were prescribed with anticoagulation combined with antiplatelet therapy; (4) major bleeding and major adverse cardiovascular events (MACE) were reported; and (5) follow-up was at least 6 months. Our exclusion criteria included observational studies, registry data, ongoing trials without results, editorials, case series, and duplicate studies (14). Previous systematic review and meta-analysis studies were screened for eligible studies.

After removing all duplicates, two reviewers independently screened titles and abstracts followed by full-text for those potentially eligible. Additionally, a third reviewer made final decisions in contested judgments.

### Outcomes

The primary safety and efficacy outcomes were major bleeding according to the criteria of Thrombolysis In Myocardial Infarction (TIMI) and trial-defined MACE, respectively. Trial-defined MACE was usually defined as a combination of either all-cause or cardiovascular mortality, myocardial infarction (MI), stroke, and stent thrombosis. The secondary safety outcomes were combined TIMI major and minor bleeding, trial-defined primary bleeding events, and intracranial hemorrhage. TIMI major bleeding was defined as any symptomatic intracranial hemorrhage, or clinically overt signs of hemorrhage (including imaging) associated with a drop in hemoglobin of ≥5 g/dL (or when the hemoglobin concentration is not available, an absolute drop in hematocrit of ≥15%). TIMI minor bleeding was defined as any clinically overt sign of hemorrhage (including imaging) that is associated with a fall in hemoglobin concentration of 3 to <5 g/dL (or, when hemoglobin concentration is not available, a fall in hematocrit of 9 to <15%). The secondary efficacy outcomes were all-cause or cardiovascular mortality, MI, stroke, stent thrombosis, and hospitalization.

### Data Extraction and Quality Assessment

Two reviewers independently extracted the following data from the eligible RCTs: Study design, baseline characteristics, interventions, and outcomes. Any contradiction was settled through consensus. All data were cross-checked. Two reviewers then used the Cochrane Collaboration tool to assess the quality of the included studies independently (15). The final author made final decisions under contradictory circumstances. Finally, all the extracted data were stored in the pre-designed excel spreadsheet.

### Data Synthesis and Analysis

We conducted a Bayesian random-effects network meta-analysis model to compare multiple regimens at the same time. We estimated odds ratios (ORs) and the associated 95% credible intervals (CrIs) for the treatment effects of the two regimens. All analyses were carried out using the *gemtc* package (version 1.0–1) (16) and the *rjags* package (version 4–11) (17) in R (version 4.0.3). We used the default setting of the package, including non-informative prior distributions, with four parallel chains. We used trace plots and Gelman–Rubin diagnostic statistics to check the convergence of Markov Chain Monte Carlo (MCMC) for all model parameters.

We calculated the rank probabilities (probability of a regimen being the best, second best, or worst for an outcome) and the surface under the cumulative ranking (SUCRA) to evaluate and rank each regimen with respect to each safety and efficacy outcome. A simple numerical summary, the supplement of the graphical display of cumulative ranking, was used to estimate the SUCRA line for each regime. SUCRA would be 1 when a regimen is certain to be the best and 0 when a regimen is certain to be the worst (18).

We assessed statistical evidence of inconsistency defined as the difference between direct and indirect comparisons of treatment effects using the node-splitting method (19).

## Results

### Search Results

**FIGURE 1:**
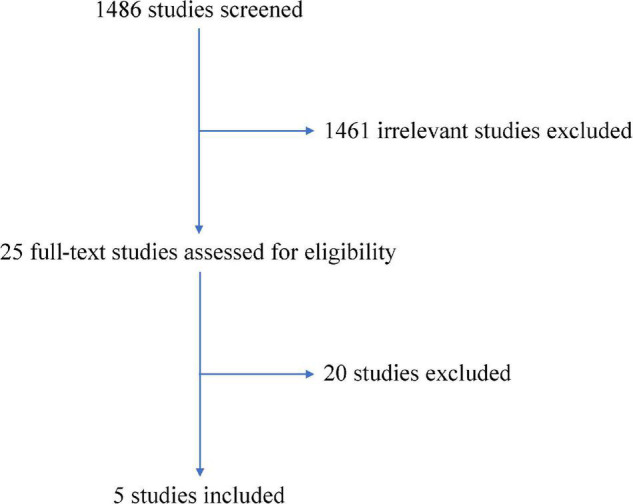
Flowchart of literature review.

Our database search yielded 1,486 unique records. Among them, 1,461 records were considered irrelevant according to the title and abstract screening. A total of 25 records were assessed in full text for eligibility. Of those, five RCTs [WOEST (7), PIONEER AF-PCI (8), RE-DUAL PCI (9), AUGUSTUS (10), and ENTRUST-AF PCI (11)] met the inclusion criteria (Figure 1). Among these RCTs, eight regimens (VKA plus P2Y12 inhibitor, VKA plus DAPT, rivaroxaban plus P2Y12 inhibitor, rivaroxaban plus DAPT, dabigatran plus P2Y12 inhibitor, apixaban plus P2Y12 inhibitor, apixaban plus DAPT, and edoxaban plus P2Y12 inhibitor) were evaluated.

### Study and Patient Characteristics

A total of 11,532 patients from the five trials were analyzed, of whom 8,426 were male. Study and patient characteristics are shown in Tables 1, 2, respectively. Except for AUGUSTUS, the follow-up time of other RCTs was more than 1 year. All RCTs were open-label, and the outcomes assessment was blinded (Supplementary Table 1).

**TABLE 1:** Study characteristics.

	WOEST	PIONEER AF-PCI	RE-DUAL PCI	AUGUSTUS	ENTRUST-AF PCI
	NCT00769938	NCT01830543	NCT02164864	NCT02415400	NCT02866175
Follow-up	12 months	12 months	14 months	6 months	12 months
Sample size	573	2,124	2,725	4,614	1,506
Enrollment	November 2008 to November 2011	May 2013 to July 2015	July 2014 to October 2016	September 2015 to April 2018	February 2017 to May 2018
Design	Open-label, randomized, controlled trial at 15 sites in the Netherlands and Belgium	International, multicenter, randomized, open-label trial at 431 sites	International, multicenter, randomized, open-label trial at 414 sites in 41 countries	Prospective, multicenter, two-by-two factorial, randomized clinical trial at 492 sites in 33 countries	Randomized, multicenter, open-label, non-inferiority phase 3b trial at 186 sites in 18 countries
Intervention	Double therapy: OAC and clopidogrel Triple therapy: aspirin, OAC, and clopidogrel	Group 1: low-dose rivaroxaban (15 mg once daily) plus a P2Y12 inhibitor for 12 months Group 2: very-low-dose rivaroxaban (2.5 mg twice daily) plus DAPT for 1, 6, or 12 months Group 3: VKA plus DAPT for 1, 6, or 12 months	Dual therapy: dabigatran (110 or 150 mg twice daily) plus a P2Y12 inhibitor (clopidogrel or ticagrelor) Triple therapy: warfarin plus DAPT (for 1–3 months)	Dual therapy: apixaban (5 mg twice daily) or VKA and a P2Y12 inhibitor for 6 months Triple therapy: apixaban or VKA and DAPT for 6 months	Dual therapy: edoxaban (60 mg once daily) plus a P2Y12 inhibitor for 12 months Triple therapy: VKA and DAPT
Inclusion criteria	A long-term indication for oral anticoagulation treatment (until at least 1 year after the study); a severe coronary lesion with indication for PCI; and age 18–80 years	Documented atrial fibrillation that occurred within 1 year before screening; patients with documented atrial fibrillation that occurred more than 1 year before screening were also eligible if the participant had been receiving oral anticoagulation for atrial fibrillation for the 3 months immediately preceding the index PCI	Men and women who were at least 18 years of age were eligible for inclusion in the trial if they had non-valvular atrial fibrillation and had successfully undergone PCI with a bare-metal or drug-eluting stent within the previous 120 h. Non-valvular atrial fibrillation could be paroxysmal, persistent, or permanent, but it could not be secondary to a reversible disorder unless long-term treatment with an oral anticoagulant was anticipated. Patients who had been receiving treatment with an oral anticoagulant before PCI and those who had not received oral anticoagulation were eligible	An age of at least 18 years; previous, persistent, permanent, or paroxysmal atrial fibrillation and planned long-term use of an oral anticoagulant; recent acute coronary syndrome or PCI; and planned use of a P2Y12 inhibitor for at least 6 months	Aged at least 18 years, and had a successful PCI for stable coronary artery disease or acute coronary syndrome
Exclusion criteria	A history of intracranial bleeding; cardiogenic shock; contra indication to use of aspirin, clopidogrel, or both; peptic ulcer in the previous 6 months; thrombocytopenia; major bleeding in the past 12 months; and pregnancy	A history of stroke or transient ischemic attack, clinically significant gastrointestinal bleeding within 12 months before randomization, a calculated creatinine clearance of less than 30 ml per minute, anemia of an unknown cause with a hemoglobin concentration of less than 10 g per deciliter, or any other condition known to increase the risk of bleeding	The presence of bioprosthetic or mechanical heart valves, severe renal insufficiency, or other major coexisting conditions	Patients who were using anticoagulation for other conditions were not eligible. Other key exclusion criteria were severe renal insufficiency, a history of intracranial hemorrhage, recent or planned coronary-artery bypass graft surgery, coagulopathy or ongoing bleeding, and contraindication to a VKA, apixaban, all P2Y12 inhibitors, or aspirin	Patients with non-valvular atrial fibrillation not secondary to a reversible disorder were included and patients with mechanical heart valves, moderate-to-severe mitral stenosis, end-stage renal disease, and other major comorbidities were excluded
Outcomes	The primary endpoint was the occurrence of any bleeding episode during 1-year follow-up. The composite secondary endpoint of death, myocardial infarction, stroke, target-vessel revascularization, and stent thrombosis	The primary safety end point was the occurrence of clinically significant bleeding. Secondary end points included the incidence of each component of the primary safety end point, as well as the following efficacy end points: the occurrence of a major adverse cardiovascular event, each component of the major adverse cardiovascular event end point, and stent thrombosis	The primary end point was a major or clinically relevant non-major bleeding event. A main secondary end point was a composite efficacy end point of thromboembolic events, death, or unplanned revascularization. Other secondary end points included a combined end point of thromboembolic events or death, as well as the individual thromboembolic events and definite stent thrombosis	The primary outcome was major or clinically relevant non-major bleeding. Secondary outcomes included death or hospitalization and a composite of ischemic events	The primary endpoint was a composite of major or clinically relevant non-major bleeding. The main efficacy outcome was the composite of cardiovascular death, stroke, systemic embolic events, myocardial infarction, and definite stent thrombosis

*VKA, vitamin K antagonist (warfarin or phenprocoumon, a target international normalization ratio of 2.0–3.0); DAPT, dual antiplatelet therapy (aspirin a P2Y12 inhibitor (clopidogrel, ticagrelor, or prasugrel).*

**TABLE 2:** Patient characteristics.

	WOEST		PIONEER AF-PCI			RE-DUAL PCI			AUGUSTUS				ENTRUST-AF PCI	
	VKA + P2Y12 inhibitor	VKA + DAPT	Rivaroxaban + P2Y12 inhibitor	Rivaroxaban + DAPT	VKA + DAPT	Dabigatran + P2Y12 inhibitor	Dabigatran + P2Y12 inhibitor	VKA + DAPT	Apixaban + DAPT	Apixaban + P2Y12 inhibitor	VKA + DAPT	VKA + P2Y12 inhibitor	Edoxaban + P2Y12 inhibitor	VKA + DAPT
Sample size	279	284	709	709	706	981	763	981	1,153	1,153	1,154	1,154	751	755
Age	70	70	70	70	70	72	69	72	71	70	71	71	69	70
Male	214	234	528	535	518	710	529	750	796	841	815	826	557	563
Diabetes, %	24.3	25.4	28.8	28.1	31.3	36.9	34.1	37.9	37.1	35.9	35.9	36.6	34.5	34.2
History, %														
MI	34.3	35.2	19.7	25.4	22.2	24.2	25,4	27,3	–	–	–	–	25	23.4
PCI	30.8	35.6	–	–	–	33.3	31.3	35.4	–	–	–	–	25	23.4
CABG	20.1	26.1	–	–	–	9.9	10.4	11.3	–	–	–	–	6.1	6.5
GB	5	4.9	1	1.3	0.7	–	–	–	–	–	–	–	–	–
CHA2DS2-VASc score, %														
<3	–	–	26.7	23.7	20.8	23.4	32.4	19.7	20.8	21.5	20.8	19.1	–	–
>2	–	–	73.3	76.3	79.2	76.6	67.6	80.3	79.2	78.5	79.2	80.9	–	–
HAS-BLED score, %														
<3	–	–	27.6	32	29.5	33.3	40.5	29.4	50.5	51.7	50.9	39.6	31.8	27
>2	–	–	72.3	68	70.5	66.8	59.5	70.6	49.5	48.3	49.1	50.4	62.2	66.6
Arterial access, %														
Radial	26.5	25	–	–	–	63	65.8	62.3	–	–	–	–	77.5	80.5
Femoral	73.1	73.2	–	–	–	36.6	33	36.8	–	–	–	–	22.4	19.3
Stent type, %														
None	1.8	1.4	0	0	0	0	0	0	–	–	–	–	–	–
Bare metal	31.9	30.3	32.6	31.2	31.8	15.2	16.1	13.6	–	–	–	–	–	–
Drug eluting	64.9	64.4	65.4	66.8	66.5	82.1	81.5	84.6	–	–	–	–	–	–
Both	1.1	3.9	2	2.9	1.7	1.9	1.3	1.2	–	–	–	–	–	–
Other	–	–	–	–	–	0.8	1	0.5	–	–	–	–	–	–
Creatinine clearance, mL/min/1.73 m^2^	–	–	78.3	77.5	80.7	76.3	83.7	75.4	78.5	79.4	78.7	80	71.8	71.7
Type of index event, %														
NSTEMI	–	–	18.5	18.3	17.8	20.7	23.5	21	–	–	–	–	21.7	20.8
STEMI	–	–	12.3	13.8	10.7	14.7	14.9	14.6	–	–	–	–	17.7	17.5
UA	–	–	20.7	21.1	23.7	19.9	16.5	16.9	–	–	–	–	14.9	16.3
Type of AF														
Persistent	–	–	20.7	20.6	21.1	17.7	17.3	18.2	–	–	–	–	18.6	19.3
Permanent	–	–	37.4	33.6	34.5	32.6	32.8	32.4	–	–	–	–	27.8	33.1
Paroxysmal	–	–	42.8	46.1	44.4	49.6	49.8	49.4	–	–	–	–	53.5	47.4

*VKA, vitamin K antagonists; MI, myocardial infarction; PCI, percutaneous coronary intervention; CABG, coronary artery bypass grafting; GB, gastrointestinal bleeding; NSTEMI, non-ST segment elevation myocardial infarction; STEMI, ST segment elevation myocardial infarction; UA, unstable angina; AF, atrial fibrillation.*

### Structure of Network Meta-Analysis

**FIGURE 2:**
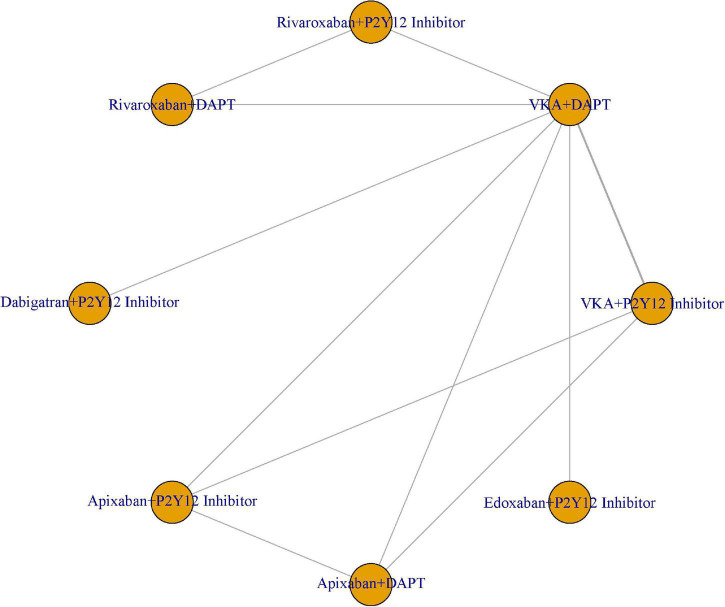
Network of eight antithrombotic treatment regimens.

We compared the eight treatment regimens at the same time: VKA plus P2Y12 inhibitor, VKA plus DAPT, rivaroxaban plus P2Y12 inhibitor, rivaroxaban plus DAPT, dabigatran plus P2Y12 inhibitor, apixaban plus P2Y12 inhibitor, apixaban plus DAPT, and edoxaban plus P2Y12 inhibitor (Figure 2). We hypothesized that the eight treatment regimens had comparable safety and efficacy. We set VKA plus P2Y12 inhibitor as a reference.

### Safety Outcomes

**FIGURE 3:**
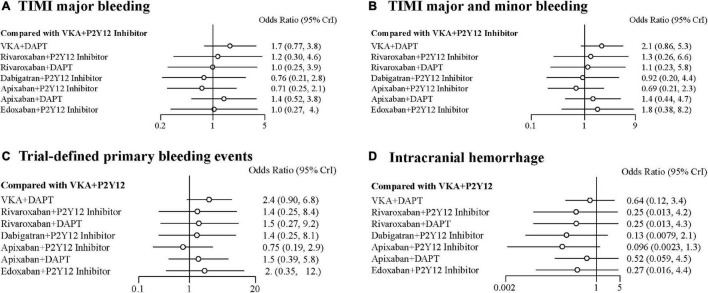
Forest plots for safety outcomes. **(A)** Thrombolysis In Myocardial Infarction (TIMI) major bleeding. **(B)** TIMI major and minor bleeding. **(C)** Trial-defined primary bleeding events. **(D)** Intracranial hemorrhage.

Compared with the ORs for VKA plus P2Y12 inhibitor, those for all safety outcomes were lower for apixaban plus P2Y12 inhibitor with no significances (Figures 3A–D). Compared with VKA plus P2Y12 inhibitor, the ORs for TIMI major bleeding were 1.70 (95% CrI, 0.77–3.80) for VKA plus DAPT, 1.20 (95% CrI, 0.30–4.60) for rivaroxaban plus P2Y12 inhibitor, 1.00 (95% CrI, 0.25–3.90) for rivaroxaban plus DAPT, 0.76 (95% CrI, 0.21–2.80) for dabigatran plus P2Y12 inhibitor, 0.71 (95% CrI, 0.25–2.10) for apixaban plus P2Y12 inhibitor, 1.40 (95% CrI, 0.52–3.80) for apixaban plus DAPT, and 1.00 (95% CrI, 0.27–4.00) for edoxaban plus P2Y12 inhibitor (Figure 3A). For combined TIMI major and minor bleeding, compared with VKA plus P2Y12 inhibitor, the ORs were 2.10 (95% CrI, 0.86–5.30) for VKA plus DAPT, 1.30 (95% CrI, 0.26–6.60) for rivaroxaban plus P2Y12 inhibitor, 1.10 (95% CrI, 0.23–5.80) for rivaroxaban plus DAPT, 0.92 (95% CrI, 0.20–4.40) for dabigatran plus P2Y12 inhibitor, 0.69 (95% CrI, 0.21–2.30) for apixaban plus P2Y12 inhibitor, 1.40 (95% CrI, 0.44–4.70) for apixaban plus DAPT, and 1.80 (95% CrI, 0.38–8.20) for edoxaban plus P2Y12 inhibitor (Figure 3B). For trial-defined primary bleeding events, compared with VKA plus P2Y12 inhibitor, the ORs were 2.40 (95% CrI, 0.90–6.80) for VKA plus DAPT, 1.40 (95% CrI, 0.25–8.40) for rivaroxaban plus P2Y12 inhibitor, 1.50 (95% CrI, 0.27–9.20) for rivaroxaban plus DAPT, 1.40 (95% CrI, 0.25–8.10) for dabigatran plus P2Y12 inhibitor, 0.75 (95% CrI, 0.19–2.90) for apixaban plus P2Y12 inhibitor, 1.50 (95% CrI, 0.39–5.80) for apixaban plus DAPT, and 2.00 (95% CrI, 0.35–12.00) for edoxaban plus P2Y12 inhibitor (Figure 3C). For intracranial hemorrhage, compared with VKA plus P2Y12 inhibitor, the ORs were 0.64 (95% CrI, 0.12–3.40) for VKA plus DAPT, 0.25 (95% CrI, 0.01–4.20) for rivaroxaban plus P2Y12 inhibitor, 0.25 (95% CrI, 0.01–4.30) for rivaroxaban plus DAPT, 0.13 (95% CrI, 0.01–4.30) for dabigatran plus P2Y12 inhibitor, 0.10 (95% CrI, 0.00–1.30) for apixaban plus P2Y12 inhibitor, 0.52 (95% CrI, 0.06–4.50) for apixaban plus DAPT, and 0.27 (95% CrI, 0.02–4.40) for edoxaban plus P2Y12 inhibitor (Figure 3D).

### Efficacy Outcomes

**FIGURE 4:**
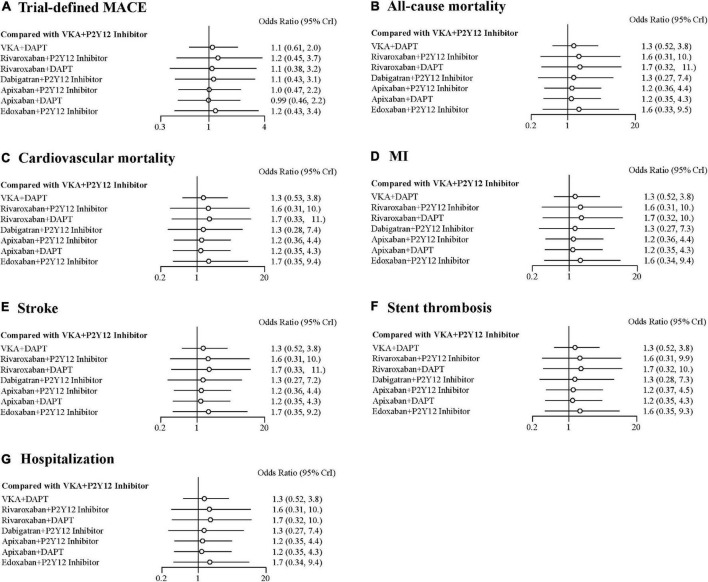
Forest plots for efficacy outcomes. **(A)** Trial-defined major adverse cardiovascular events (MACE). **(B)** All-cause mortality. **(C)** Cardiovascular mortality. **(D)** Myocardial infarction (MI). **(E)** Stroke. **(F)** Stent thrombosis. **(G)** Hospitalization.

There were no statistical differences among the antithrombotic regimens in trial-defined MACE, all-cause or cardiovascular mortality, MI, stroke, stent thrombosis, and hospitalization (Figures 4A–G). For trial-defined MACE, compared with VKA plus P2Y12 inhibitor, the ORs were 1.10 (95% CrI, 0.61–2.00) for VKA plus DAPT, 1.20 (95% CrI, 0.45–3.70) for rivaroxaban plus P2Y12 inhibitor, 1.10 (95% CrI, 0.38–3.20) for rivaroxaban plus DAPT, 1.10 (95% CrI, 0.43–3.10) for dabigatran plus P2Y12 inhibitor, 1.00 (95% CrI, 0.47–2.20) for apixaban plus P2Y12 inhibitor, 0.99 (95% CrI, 0.46–2.20) for apixaban plus DAPT, and 1.20 (95% CrI, 0.43–3.40) for edoxaban plus P2Y12 inhibitor (Figure 4A). For all-cause mortality, compared with VKA plus P2Y12 inhibitor, the ORs were 1.30 (95% CrI, 0.52–3.80) for VKA plus DAPT, 1.60 (95% CrI, 0.31–10.00) for rivaroxaban plus P2Y12 inhibitor, 1.70 (95% CrI, 0.32–11.00) for rivaroxaban plus DAPT, 1.30 (95% CrI, 0.27–7.40) for dabigatran plus P2Y12 inhibitor, 1.20 (95% CrI, 0.36–4.40) for apixaban plus P2Y12 inhibitor, 1.20 (95% CrI, 0.35–4.30) for apixaban plus DAPT, and 1.60 (95% CrI, 0.33–9.50) for edoxaban plus P2Y12 inhibitor (Figure 4B). For cardiovascular mortality, compared with VKA plus P2Y12 inhibitor, the ORs were 1.30 (95% CrI, 0.53–3.80) for VKA plus DAPT, 1.60 (95% CrI, 0.31–10.00) for rivaroxaban plus P2Y12 inhibitor, 1.70 (95% CrI, 0.33–11.00) for rivaroxaban plus DAPT, 1.30 (95% CrI, 0.28–7.40) for dabigatran plus P2Y12 inhibitor, 1.20 (95% CrI, 0.36–4.40) for apixaban plus P2Y12 inhibitor, 1.20 (95% CrI, 0.35–4.30) for apixaban plus DAPT, and 1.70 (95% CrI, 0.35–9.40) for edoxaban plus P2Y12 inhibitor (Figure 4C). For MI, compared with VKA plus P2Y12 inhibitor, the ORs were 1.30 (95% CrI, 0.52–3.80) for VKA plus DAPT, 1.60 (95% CrI, 0.31–10.00) for rivaroxaban plus P2Y12 inhibitor, 1.70 (95% CrI, 0.32–10.00) for rivaroxaban plus DAPT, 1.30 (95% CrI, 0.27–7.30) for dabigatran plus P2Y12 inhibitor, 1.20 (95% CrI, 0.36–4.40) for apixaban plus P2Y12 inhibitor, 1.20 (95% CrI, 0.35–4.30) for apixaban plus DAPT, and 1.60 (95% CrI, 0.34–9.40) for edoxaban plus P2Y12 inhibitor (Figure 4D). The details of stroke, stent thrombosis, and hospitalization are shown in Figures 4E–G.

### Ranking of Antithrombotic Regimens

**FIGURE 5:**
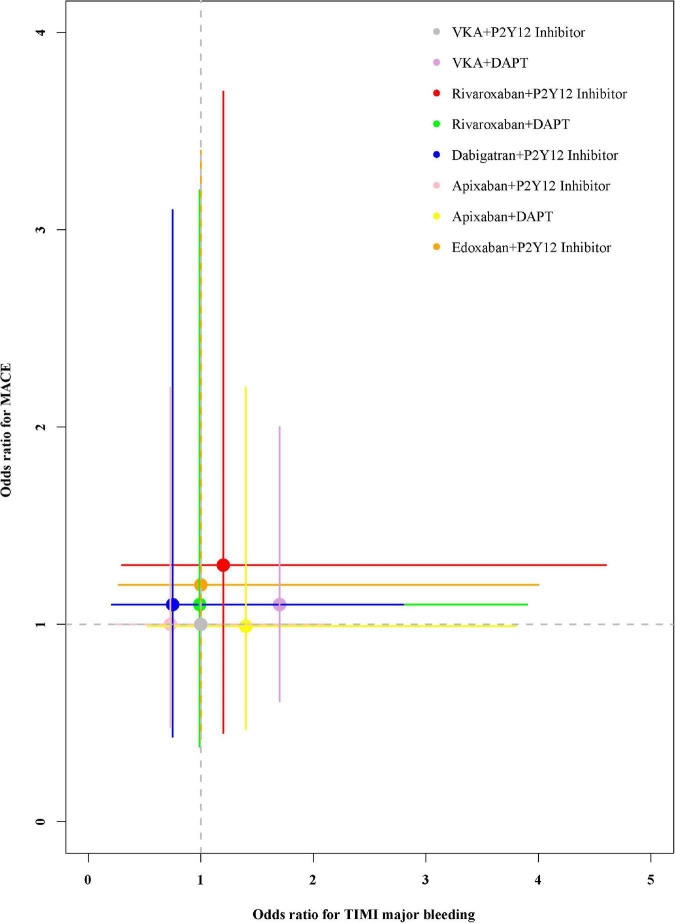
Odds ratios for TIMI major bleeding and trial-defined MACE.

**FIGURE 6:**
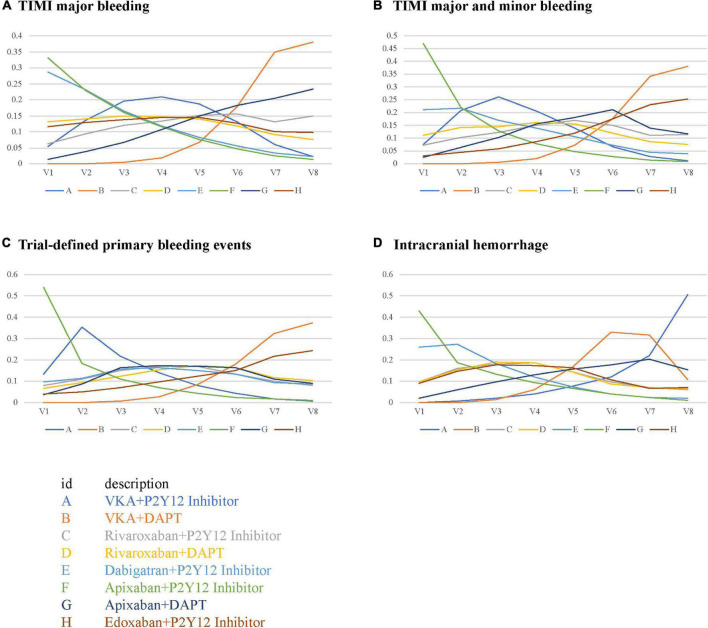
Rank probabilities for safety outcomes. **(A)** TIMI major bleeding. **(B)** TIMI major and minor bleeding. **(C)** Trial-defined primary bleeding events. **(D)** Intracranial hemorrhage.

**FIGURE 7:**
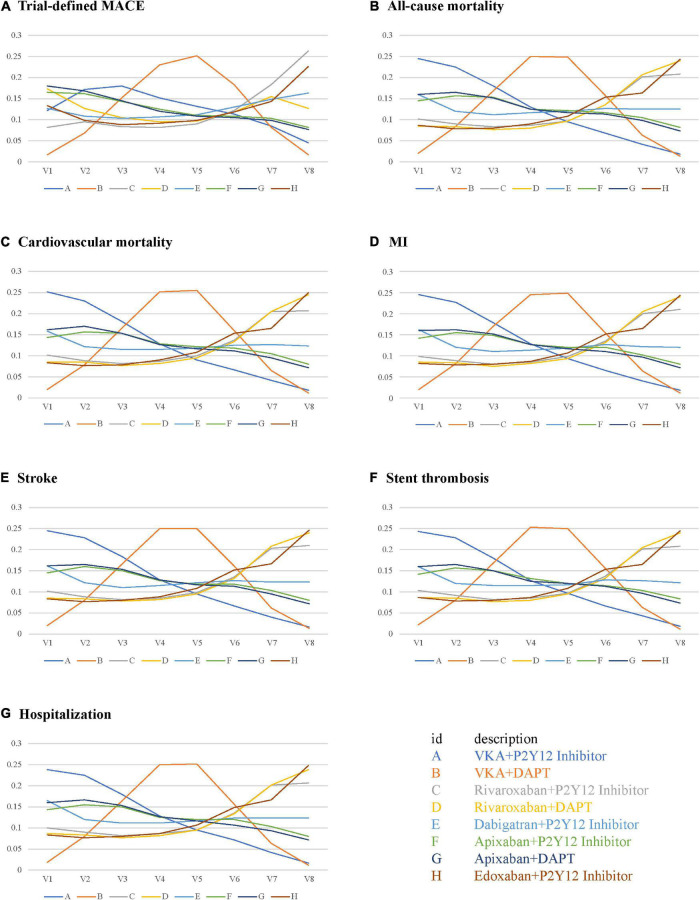
Rank probabilities for efficacy outcomes. **(A)** Trial-defined MACE. **(B)** All-cause mortality. **(C)** Cardiovascular mortality. **(D)** MI. **(E)** Stroke. **(F)** Stent thrombosis. **(G)** Hospitalization.

The performance of the tested regimens was visualized in a two-dimensional forest plot of ORs (Figure 5). Apixaban plus P2Y12 inhibitor was the highest-ranking of safety outcomes (SUCRA values of TIMI major bleeding, combined TIMI major and minor bleeding, trial-defined primary bleeding events, and intracranial hemorrhage were 0.331850, 0.469975, 0.539425, and 0.4321875, respectively) (Figure 6). Apixaban plus DAPT was the highest-ranking of trial-defined MACE (SUCRA value was 0.180525) (Figure 7A). VKA plus P2Y12 inhibitor was the highest-ranking of all-cause mortality, cardiovascular mortality, MI, stroke, stent thrombosis, and hospitalization (SUCRA values were 0.2439375, 0.2508375, 0.2455375, 0.24465, 0.24215, and 0.23865, respectively) (Figures 7B–G). All fitted models were well converged (Supplementary Figure 1) and we found no evidence of statistical inconsistency in our Bayesian network meta-analysis (Supplementary Figure 2).

## Discussion

Antithrombotic drugs are widely used treatments for patients with AF undergoing PCI worldwide. In this comprehensive Bayesian network meta-analysis of five multicenter RCTs, we investigated the safety and efficacy profile of eight antithrombotic regimens in 11,532 AF patients undergoing PCI. Apixaban plus P2Y12 inhibitor seems to reduce bleeding complications while maintaining antithrombotic efficacy. Moreover, for most efficacy indicators, VKA plus P2Y12 inhibitor ranking is still very high. The summary of this study indicated that discontinuing aspirin seems to be feasible clinically, which is consistent with a previous study (12).

Most guideline recommendations in cardiology are based on low-quality evidence, and the field of antithrombotic therapy for AF undergoing PCI is no exception. In the past few years, we have been hovering directly between dual antiplatelet therapy and three antiplatelet therapy (20). Here, to our knowledge, we determined the specific drug composition for the first time. This is due to the publication of high-quality evidence, which allows us to more objectively evaluate the risk of bleeding and ischemia.

ACTIVE W indicated that oral anticoagulation therapy is superior to clopidogrel plus aspirin for the prevention of vascular events in patients with AF at high risk of stroke, especially in those already taking oral anticoagulation therapy (21). The prognosis is unsatisfactory in warfarin-treated stented patients, and warfarin plus aspirin increased the risk of stent thrombosis (22). Oral anticoagulants exert antiplatelet effects to prevent stroke and recurrent MI, and further synergize through P2Y12 inhibition (23, 24). Therefore, oral anticoagulants plus P2Y12 inhibition may be appropriate for the vast majority of patients with AF undergoing PCI. NOACs simplify long-term anticoagulation therapy by promoting optimal thromboembolic protection and reducing bleeding complications (20). Our results of rank probabilities and SUCRA indicated that apixaban plus P2Y12 inhibitor had the least TIMI major bleeding, combined TIMI major and minor bleeding, trial-defined primary bleeding events, and intracranial hemorrhage, indicating apixaban plus P2Y12 inhibitor is the safest, and that VKA plus P2Y12 inhibitor had the least all-cause mortality, cardiovascular mortality, MI, stroke, stent thrombosis, and hospitalization, indicating VKA plus P2Y12 inhibitor is the most effective.

This is a study-level meta-analysis without obtaining individual patient data, which has well-known inherent limitations. In addition, due to the limited number of studies (less than 10), we were unable to identify potential publication bias (25). Third, our primary efficacy outcome is trial-defined MACE, but because each RCT defines MACE differently, our conclusion about trial-defined MACE may not be entirely credible. We concluded here that apixaban plus DAPT had the highest SUCRA value than others, which may be inconsistent with our previous knowledge. Moreover, our results are primarily on the clopidogrel-based therapy (most patients received this P2Y12 inhibitor), therefore, whether the use of strategies to identify poor-responders or the use of alternative P2Y12 inhibitors may reduce the risk of thrombus while retaining the bleeding benefit remains to be investigated. Finally, future studies focused on individual patient-level data analyses, whether trial-specific or pooled, may help to further refine which patients would benefit most from longer-term apixaban plus P2Y12 inhibitor consumption.

## Conclusion

Apixaban plus P2Y12 inhibitor seems to be linked with fewer bleeding complications while retaining antithrombotic efficacy. Moreover, the ranking of VKA plus P2Y12 inhibitor is highest for most efficacy indicators.

## Data Availability Statement

The original contributions presented in the study are included in the article/Supplementary Material, further inquiries can be directed to the corresponding author/s.

## Author Contributions

BL and NG designed the research. BL wrote the manuscript. NG revised the manuscript. All authors acquired and analyzed the data, interpreted the results, and read and approved the final manuscript.

## Conflict of Interest

The authors declare that the research was conducted in the absence of any commercial or financial relationships that could be construed as a potential conflict of interest.

## Publisher’s Note

All claims expressed in this article are solely those of the authors and do not necessarily represent those of their affiliated organizations, or those of the publisher, the editors and the reviewers. Any product that may be evaluated in this article, or claim that may be made by its manufacturer, is not guaranteed or endorsed by the publisher.

## Supplementary Material

Supplementary Figure 1 Model diagnostics under the assumption of evidence consistency. **(A)** TIMI major bleeding. **(B)** TIMI major and minor bleeding. **(C)** Trial-defined primary bleeding events. **(D)** Intracranial hemorrhage. **(E)** Trial-defined MACE. **(F)** All-cause mortality. **(G)** Cardiovascular mortality. **(H)** MI. **(I)** Stroke. **(J)** Stent thrombosis. **(K)** Hospitalization.

Supplementary Figure 2 Checking the assumption of evidence inconsistency.
