# Supplementary material for: Comparative Safety and Efficacy of Eight Antithrombotic Regimens for Patients With Atrial Fibrillation Undergoing Percutaneous Coronary Intervention
Source: Front Cardiovasc Med. 2022 Mar 21;9:832164. doi: 10.3389/fcvm.2022.832164 (PMC8978794; doi:10.3389/fcvm.2022.832164)
Supplement: Supplementary file 1 [file Table_1.DOCX]

Table S1. Risk of bias of included studies

|  | WOEST | PIONEER AF‐PCI | RE‐DUAL PCI | AUGUSTUS | ENTRUST-AF PCI |
| --- | --- | --- | --- | --- | --- |
| Selection bias | ⊕ | ⊕ | ⊕ | ⊕ | ⊕ |
| Performance bias^1^ | ⊕ | ⊕ | ⊕ | ⊕ | ⊕ |
| Attrition bias^2^ | ⊕ | ⊕ | ⊕ | ⊕ | ⊕ |
| Reporting bias | ⊕ | ⊕ | ⊕ | ⊕ | ⊕ |
| Other source of bias | ⊕ | ⊕ | ⊕ | ⊕ | ⊕ |

Note: ^1^ Despite that the used an open label trial design, the outcome assessment was blinded, and therefore the study design did not influence reported outcomes and hereby negatively effecting the quality of evidence. ^2^ All studies reported minimal loss to follow-up.
