# Supplementary material for: Comparative Safety and Efficacy of Eight Antithrombotic Regimens for Patients With Atrial Fibrillation Undergoing Percutaneous Coronary Intervention
Source: Front Cardiovasc Med. 2022 Mar 21;9:832164. doi: 10.3389/fcvm.2022.832164 (PMC8978794; doi:10.3389/fcvm.2022.832164)

A. TIMI major bleeding.

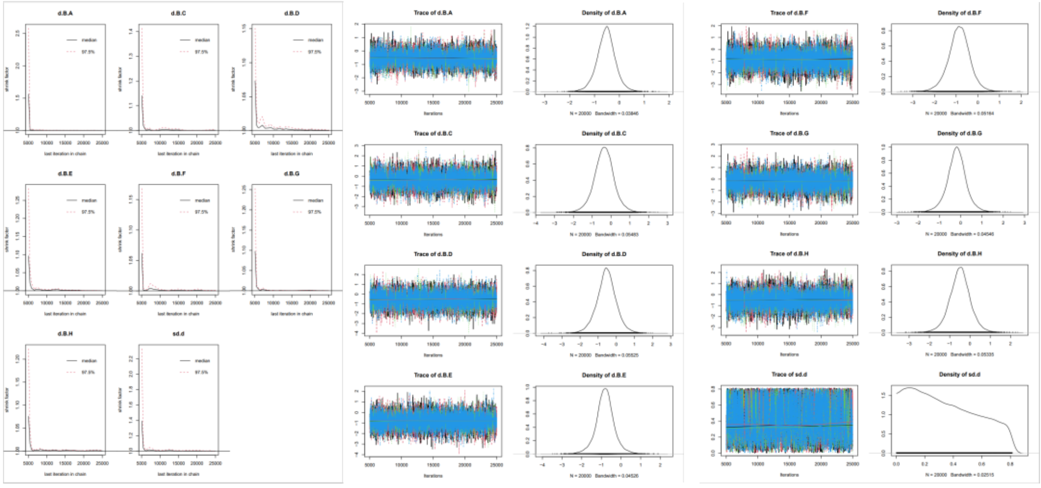

B. TIMI major and minor bleeding.

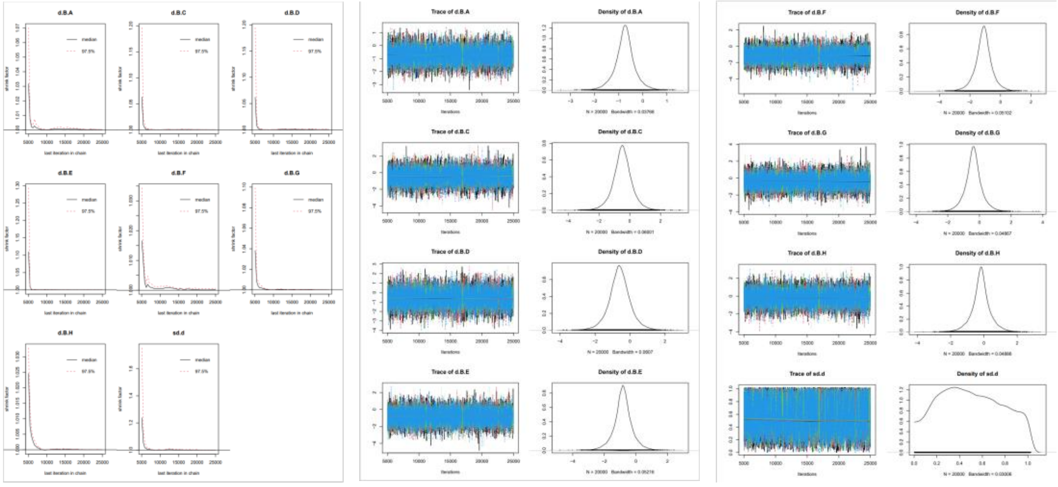

C. Trial-defined primary bleeding events.

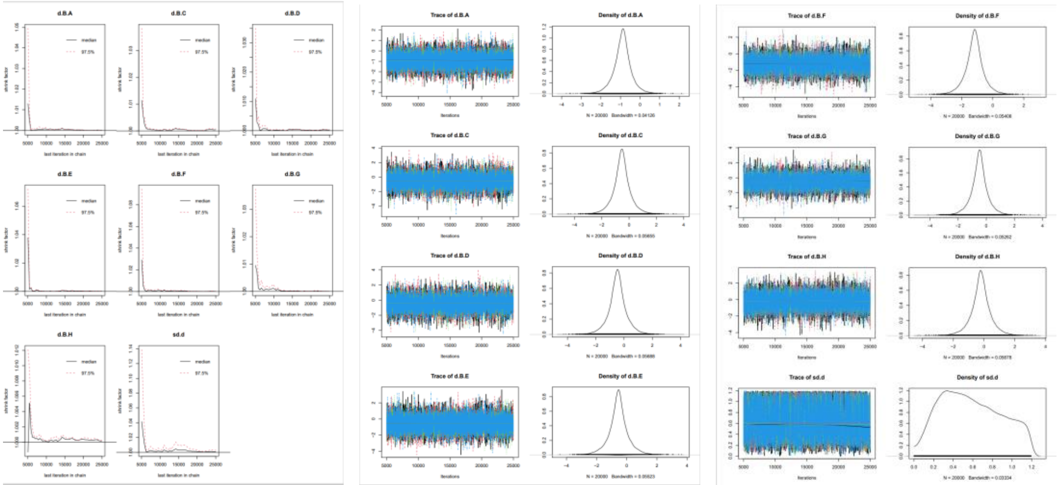

D. Intracranial hemorrhage.

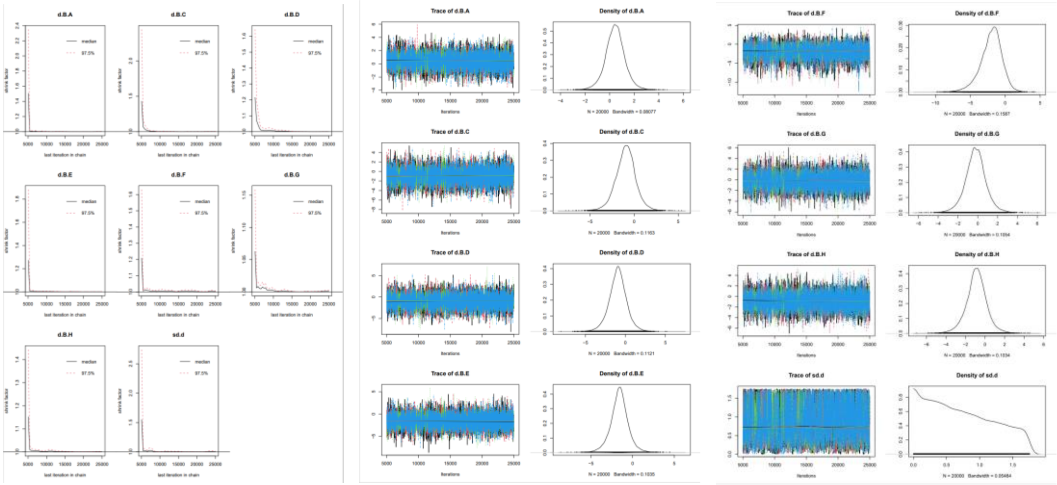

E. Trial-defined MACE.

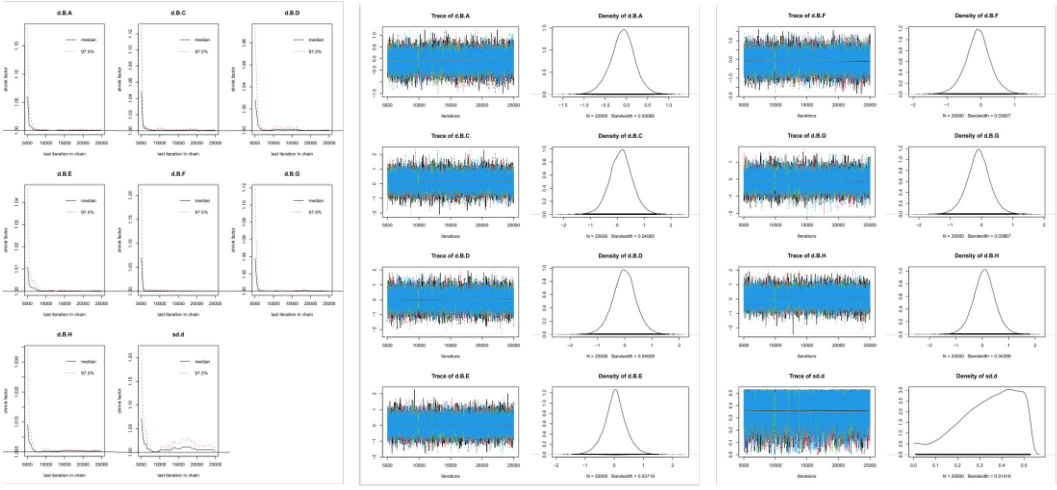

F. All-cause mortality.

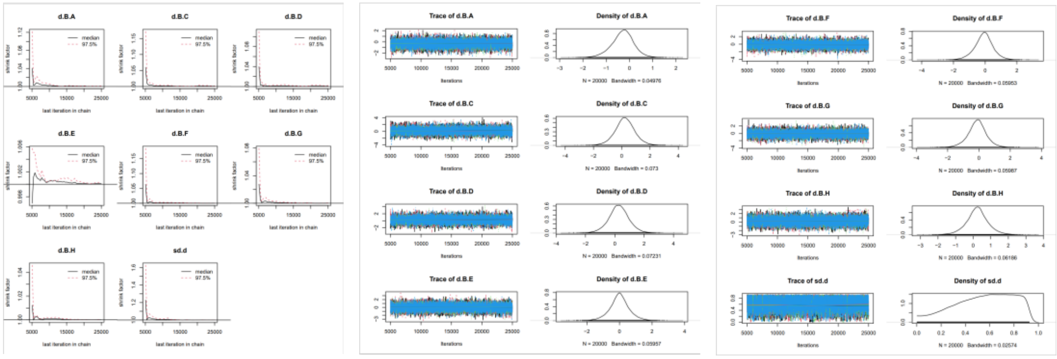

G. Cardiovascular mortality.

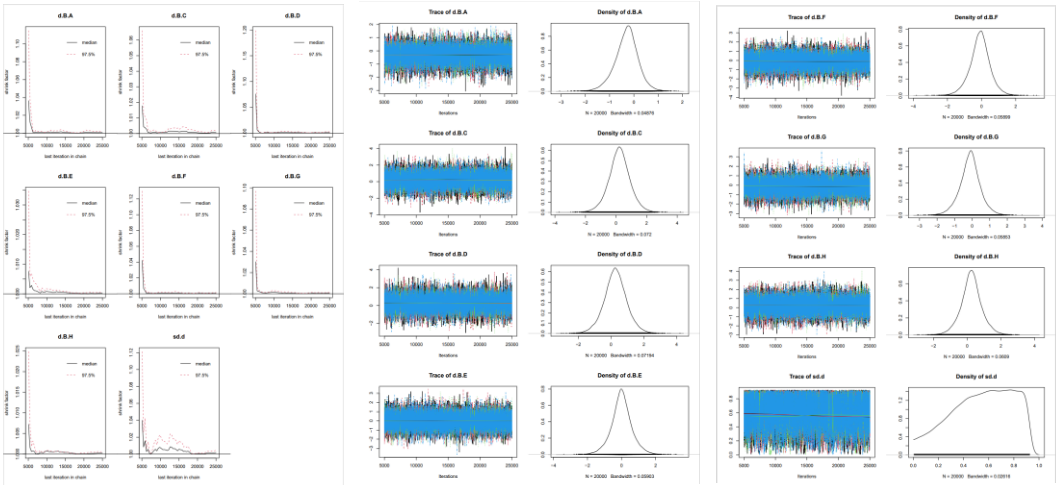

H. MI.

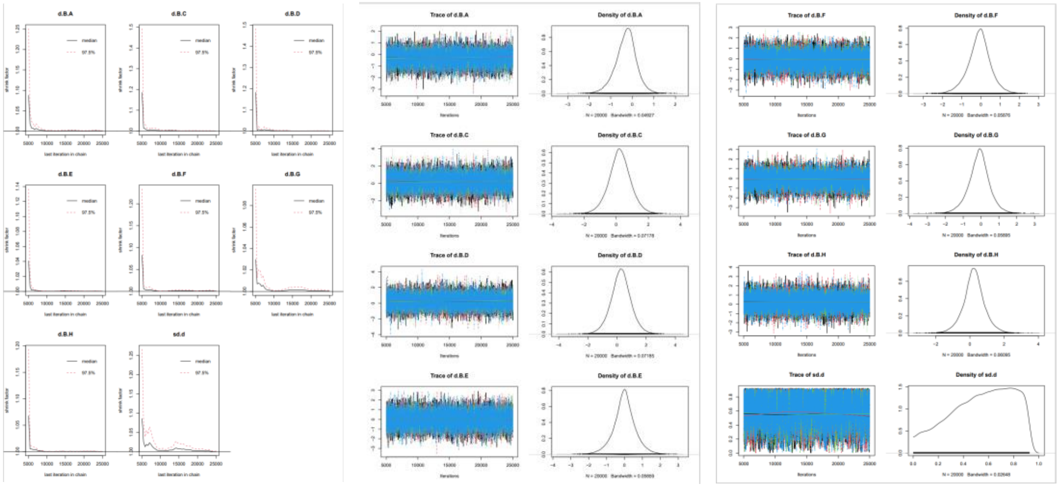

I. Stroke.

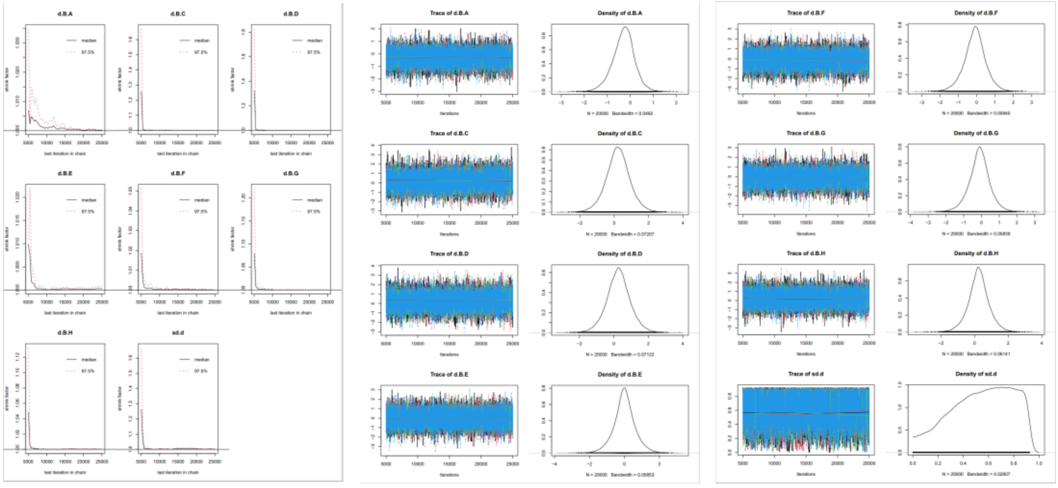

J. Stent thrombosis.

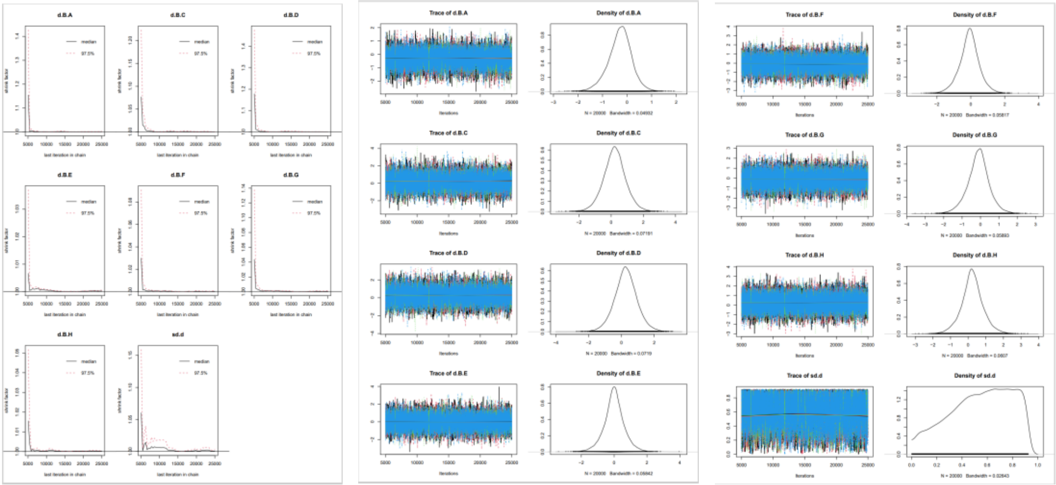

K. Hospitalization.

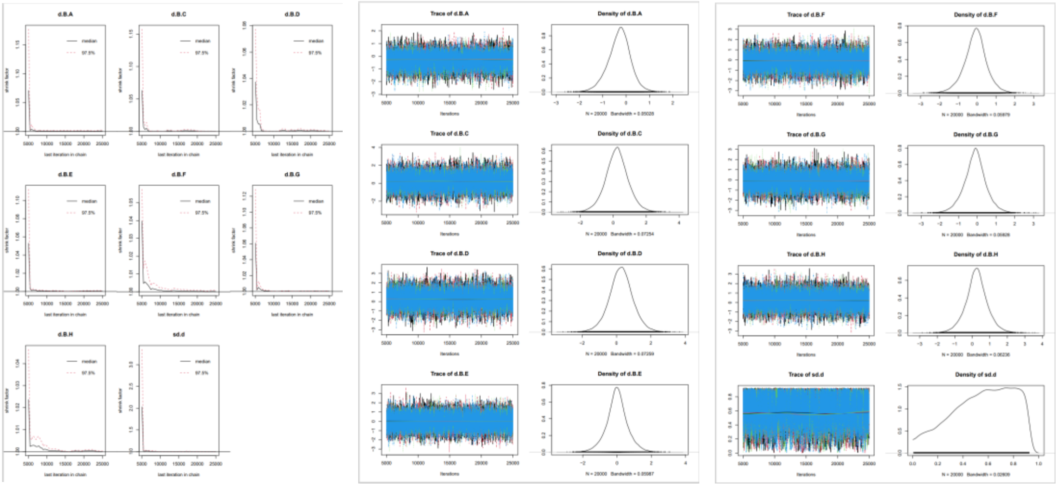

Supplement: Supplementary Figure 1 — Model diagnostics under the assumption of evidence consistency. (A) TIMI major bleeding. (B) TIMI major and minor bleeding. (C) Trial-defined primary bleeding events. (D) Intracranial hemorrhage. (E) Trial-defined MACE. (F) All-cause mortality. (G) Cardiovascular mortality. (H) MI. (I) Stroke. (J) Stent thrombosis. (K) Hospitalization. [file Data_Sheet_1.PDF]
