# Supplementary figures and images for: Comparative Safety and Efficacy of Eight Antithrombotic Regimens for Patients With Atrial Fibrillation Undergoing Percutaneous Coronary Intervention
Source: Front Cardiovasc Med. 2022 Mar 21;9:832164. doi: 10.3389/fcvm.2022.832164 (PMC8978794; doi:10.3389/fcvm.2022.832164)

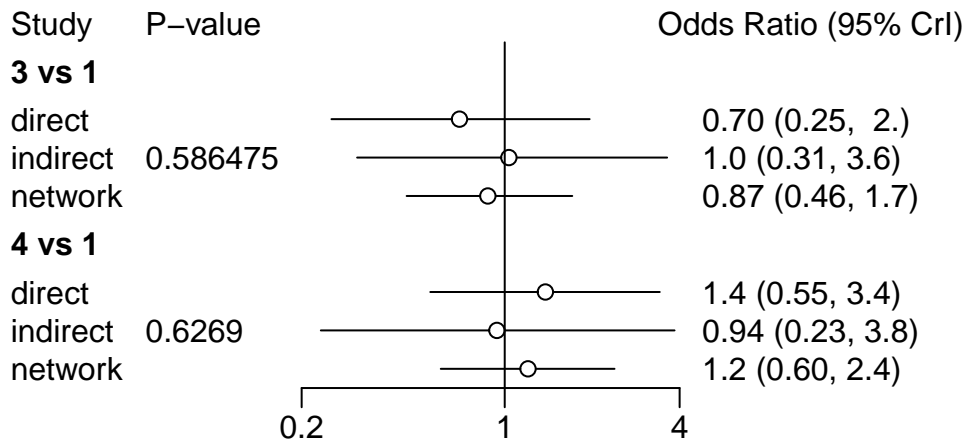

Supplement: Supplementary Figure 2 — Checking the assumption of evidence inconsistency. [file Data_Sheet_2.PDF]
